# The dynamics of methicillin-resistant *Staphylococcus aureus* exposure in a hospital model and the potential for environmental intervention

**DOI:** 10.1186/1471-2334-13-595

**Published:** 2013-12-17

**Authors:** Nottasorn Plipat, Ian H Spicknall, James S Koopman, Joseph NS Eisenberg

**Affiliations:** 1 Department of Epidemiology, School of Public Health, University of Michigan, Ann Arbor, Michigan, USA

## Abstract

**Background:**

Methicillin-resistant *Staphylococcus aureus* (MRSA) is a major cause of healthcare-associated infections. An important control strategy is hand hygiene; however, non-compliance has been a major problem in healthcare settings. Furthermore, modeling studies have suggested that the law of diminishing return applies to hand hygiene. Other additional control strategies such as environmental cleaning may be warranted, given that MRSA-positive individuals constantly shed contaminated desquamated skin particles to the environment.

**Methods:**

We constructed and analyzed a deterministic environmental compartmental model of MRSA fate, transport, and exposure between two hypothetical hospital rooms: one with a colonized patient, shedding MRSA; another with an uncolonized patient, susceptible to exposure. Healthcare workers (HCWs), acting solely as vectors, spread MRSA from one patient room to the other.

**Results:**

Although porous surfaces became highly contaminated, their low transfer efficiency limited the exposure dose to HCWs and the uncolonized patient. Conversely, the high transfer efficiency of nonporous surfaces allows greater MRSA transfer when touched. In the colonized patient’s room, HCW exposure occurred more predominantly through the indirect (patient to surfaces to HCW) mode compared to the direct (patient to HCW) mode. In contrast, in the uncolonized patient’s room, patient exposure was more predominant in the direct (HCW to patient) mode compared to the indirect (HCW to surfaces to patient) mode. Surface wiping decreased MRSA exposure to the uncolonized patient more than daily surface decontamination. This was because wiping allowed higher cleaning frequency and cleaned more total surface area per day.

**Conclusions:**

Environmental cleaning should be considered as an integral component of MRSA infection control in hospitals. Given the previously under-appreciated role of surface contamination in MRSA transmission, this intervention mode can contribute to an effective multiple barrier approach in concert with hand hygiene.

## Background

Methicillin-resistant *Staphylococcus aureus* (MRSA) has become an important cause of healthcare-associated infections in the U.S. and worldwide [1]–[3]. An important strategy for preventing healthcare-associated infection (HAI), including MRSA infections, has been hand hygiene [4]–[8]. The role of hand hygiene in decreasing HAIs was established in the 1840 s, when Semmelweis demonstrated that hand scrubbing with chlorinated lime solution resulted in a drastic decrease in maternal mortality due to puerperal sepsis [9]. A study in 1962, also showed that newborns, cared for by nurses who did not perform hand hygiene, were more likely to acquire *S. aureus* infection than those cared for by nurses who performed hand hygiene [10]. Although these historical studies as well as many intervention studies have demonstrated the efficacy of hand hygiene, the evidence of benefit has not always been translated into routine practice [11,12]. Hand hygiene noncompliance remains a major problem in healthcare settings [11]. Furthermore, studies have suggested that the law of diminishing return applies to hand hygiene, with the greatest benefits occurring in the first 20% of compliance. The additional benefits of hand hygiene decrease as the baseline compliance levels increase [13,14].

Prevention of MRSA infections will likely need more than hand hygiene intervention; this is partly because of poor compliance, and also because of possibility of recontamination of hands from surface as well as cross-contamination from the skin [15]–[19]. Due to these limitations, interventions performed in concert with hand hygiene may be important to further decrease MRSA HAIs. Example of a broad class of intervention includes environmental cleaning. Environmental cleaning is aimed at removing or inactivating pathogens in the environment [20]. Generally, hospital surface decontamination is performed by environmental health personnel, who manually apply liquid disinfectant to the surfaces on a regular basis. Another method of environmental cleaning is surface wiping. This can be done by anyone including healthcare workers by wiping a surface immediately after touching it. Surface wiping relies on the mechanical removal of contamination, thus, does not require a strong microbicidal formulation [21].

Generally, colonized or infected individuals are the sources of MRSA in the environment. Approximately 10-30% of the general population has *S. aureus* colonization on their skin or in their noses [22]. Studies conducted in the 1960s revealed that *S. aureus* is not freely suspended in air or on surfaces, but rather carried by desquamated epithelial cells [23,24]. As many as 10^6^ to 10^7^ cells, with dimension ranging from 8 to 20 μm, can be dispersed from the body in 24 h. These aerial epithelial cells settle onto surfaces, but they may become temporarily re-aerosolized when they are mechanically disturbed, only to redeposit back to the surfaces [25]. Huang et al found that patients, admitted to rooms previously occupied by MRSA-positive patients, had increased odds of subsequent MRSA acquisition [26]. However, the mechanisms describing how shedding from one patient leads to exposure and acquisition in another are yet to be well characterized. Possible exposure pathways include direct skin-to-skin contact and indirect environmental spread via contaminated environmental surfaces.

Healthcare workers (HCWs) play important roles in the transmission of healthcare-associated pathogens such as MRSA. HCWs colonized with MRSA, although rarely reported, have been shown to be important sources of infections that have led to outbreaks [17]. Additionally, HCWs have been implicated as mechanical vectors of transmission between patients [17]. A few modeling studies examined the importance of HCWs as vectors [27,28], but did not explicitly include the environment. Other models that explicitly incorporate the environment have examined control strategies and the key interactions among patients, HCWs, and their environment [29]–[31]. Considering these studies, we have focused on a more comprehensive exposure model that explicitly accounts for fate and transport processes and provides a platform to investigate the effects of environmental interventions.

Specifically, we developed an exposure assessment model where MRSA is continuously shed from a colonized patient into the environment and is spread through HCWs’ hands acting as vectors to another patient. The model includes one colonized patient, one uncolonized patient, and HCWs who care for them. The analysis was focused on MRSA exposure pathways to HCWs and the uncolonized patient. Direct MRSA exposure to the HCW was quantified by the net flow of MRSA resulting from the skin-to-skin contact with the colonized patient, while indirect exposure was quantified by the net flow of MRSA due to contamination of two surface types in the room, porous and nonporous. We employed similar procedures to quantify both direct as well as indirect exposure to the uncolonized patient.

## Methods

### Model overview

We constructed and analyzed a deterministic compartmental model of MRSA fate and transport between two hypothetical hospital rooms. In one room, there is a colonized patient, who is the only MRSA source in the system. A colonized patient is defined as an individual who harbors MRSA on the skin and in the nose, both of which produces a constant level of MRSA. This colonized patient sheds MRSA onto environmental surfaces both by: (1) aerial dispersal of MRSA-contaminated desquamated epithelial cells that eventually get deposited onto a surface; and (2) the touching of the surface with contaminated hands. HCWs visit the colonized patient’s room hourly. While in the room, HCW’s hands become contaminated by touching these contaminated surfaces and by contact with the colonized patient.

In the second room, there is an uncolonized patient that may become exposed either by touching contaminated surfaces or by contact with HCWs. The HCW’s contaminated hands are the only sources of MRSA in this room. HCWs were not colonized with MRSA and do not shed MRSA; instead, they served as vectors of the transmission process. We assume that each HCW works an eight-hour shift. Each hour of a shift is organized identically as follows: The HCW visits the colonized patient’s room during the first 20 minutes, the uncolonized patient’s room during the next 20 minutes, and the HCWs’ station during the last 20 minutes. At the HCW’s station, we assume that there is no surface touching, thus, no MRSA transfer. This cycle is repeated every hour throughout the eight-hour shift. During each room visit, the HCW touches the patient and the two environmental surfaces at specified touching rates (τ_hcw-pt_ and τ_hcw-sf_, respectively). Patients also touch the surfaces at a specified touching rate (τ_pt-sf_).

**Figure 1:**
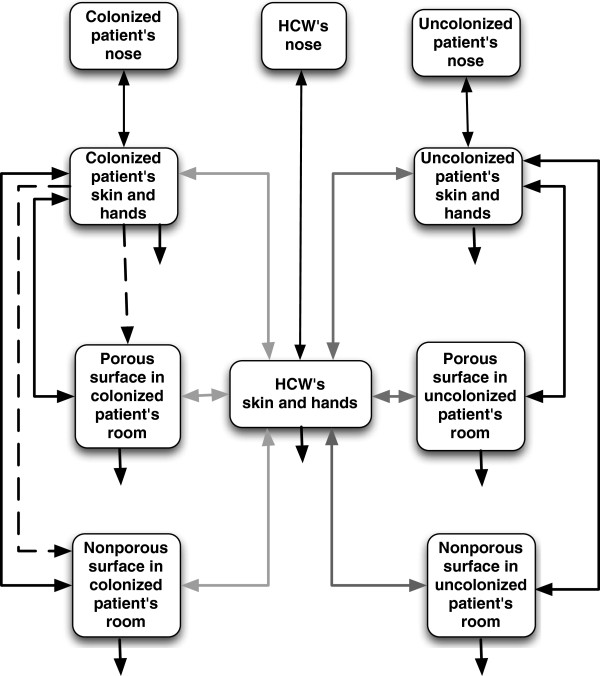
**Diagram of our 10 compartments fate and transport exposure model.** Solid arrows represent pathogen flows due to touching events or natural die off. Dashed arrows represent shedding from colonized patient to the porous and nonporous surfaces in the room. Black arrows represent time independent flows within the colonized and uncolonized patient’s room, and touching noses. Grey arrows represent time dependent flows to and from HCW’s skin and hands during the first 20 minutes of the hour (light grey) and during the next 20 minutes of the hour (dark grey).

Our model is an ordinary differential equation-based model consisting of 10 compartments as shown in Figure 1. These compartments track the number of pathogens in the following locations: (1) on the colonized patient’s skin, (2) on the uncolonized patient’s skin, (3) on the HCW’s skin, (4) on the porous surfaces in the colonized patient’s room, (5) on the porous surfaces in the uncolonized patient’s room, (6) on the nonporous surfaces in the colonized patient’s room, (7) on the nonporous surfaces in the uncolonized patient’s room, (8) in the uncolonized patient’s nose, (9) in the HCW’s nose, and (10) in the colonized patient’s nose. The nose of a colonized patient, one of the sources of MRSA, is assumed to be at steady state with constant MRSA contamination levels. However, the uncolonized patient and the uncolonized HCWs did not have MRSA in their noses at the start of the simulation; their noses became contaminated with MRSA once their hands touch the noses. Each compartment representing presence of MRSA on the skin of either patients or HCWs is composed of two components, namely, “hand-skin” and “exposed non-hand-skin,” and those are homogeneously mixed. Touching rates and transfer efficacies are associated only with the hand-skin, a proportional component of the skin compartment. MRSA concentration within each compartment is assumed to be instantaneously and homogenously spread.

### Model description

MRSA shedding to the environment upon admission of a colonized patient occurs through two pathways: (1) continuous MRSA dispersal into the air on desquamated epithelial cells (α) and (2) intermittent MRSA transfer from the colonized patient’s nose to hands (ρ_n_) to environmental surfaces (ρ_p_, ρ_np_). MRSA in the air is assumed to be instantaneously settled onto environmental surfaces. Once transferred onto environmental surfaces (or skin and hands), some MRSA might naturally die off (μ_sk_, μ_p_, μ_np_). When HCWs and patients touch these contaminated surfaces, some fraction of MRSA might be picked up. This fraction depends on the transfer efficiency (ρ_p_, ρ_np_) and the MRSA quantity on the surfaces and skin. Once MRSA is transferred onto the skin of either patients or HCWs, it might be redeposited back to surfaces (ρ_p_, ρ_np_). Finally, MRSA on the skin might be inoculated to the nose, when individuals touch their noses with their contaminated hands.

Table 1 presents model parameters. The Additional file 1 provides the justification for the parameter values used in our model, while the Additional file 2 provides the equations used in the model.

**Table 1:** Model parameters and their values

	**Parameter**	**Value**
**Shedding parameters:**		
Shedding (air dispersal) rate (cfu/cm^2^/min)	α	0.01
**Survival parameters:**		
Die-off rate on skin and hand (min^-1^)	μ_sk_	0.00353
Die-off rate on porous surface (min^-1^)	μ_p_	0.000632
Die-off rate on nonporous surface (min^-1^)	μ_np_	0.0002
**Contact parameters:**		
Rate of patient touching surfaces (min^-1^)	τ_pt-sf_	0.134
Rate of HCW touching patient (min^-1^)	τ_hcw-pt_	0.4
Rate of HCW touching surfaces (min^-1^)	τ_hcw-sf_	0.4
Rate of touching the nose (min^-1^)	τ_n_	0.025
Rate of HCW wiping nonporous surface (min^-1^)	ω_np_	0.4
**Transfer efficiency parameters:**		
Transfer efficiency from porous surface to hand	ρ_p_	0.1
Transfer efficiency from nonporous surface to hand	ρ_np_	0.4
Transfer efficiency from hand to skin	ρ_sk_	0.35
Transfer efficiency from fingertip to nose	ρ_n_	0.2
**Surface area parameters:**		
Total exposed skin and hand surface area of patients (cm^2^)	A_pt_	2000
Total exposed skin and hand surface area of HCWs (cm^2^)	A_hcw_	2000
Total porous surface area (cm^2^)	A_p_	2000
Total nonporous surface area (cm^2^)	A_np_	2000
Nose surface area (cm^2^)	A_n_	4
Hand contact surface area (cm^2^)	A_h_	150
Fingertip contact surface area (cm^2^)	A_f_	1
**Interventions:**		
Daily surface decontamination efficacy	ϵ_d_	0, 50, 100%
Wiping efficacy	ϵ_w_	0, 50, 100%

### Surface touching event

Surface touching is the event that transfers MRSA from one compartment to another. For each touching event, there are bidirectional flows of MRSA to and from the two contacting surfaces. The net quantity of these two bidirectional flows determines whether a contacting surface will be the net-giver or the net-receiver, thus determining when the resultant event is the contamination of the touched surface or exposure to the patients/HCWs.

In this model, we compared two exposure pathways in the two patient’s rooms: (1) direct and indirect exposure to the HCWs in the colonized patient’s room, and (2) direct and indirect exposure to the uncolonized patient’s room. Direct exposure resulted from the skin-to-skin contact between the HCWs and the patients. Indirect exposure resulted from the skin-to-surface-to-skin contact.

In addition to the direct and indirect exposures, HCWs and patients touched their noses with their fingertips. For the colonized patient, this type of contact led to hand contamination, which later contaminated surfaces when the patient touched the surfaces. For the uncolonized patient and the HCWs, touching noses resulted in increase in MRSA concentration in their noses, but did not lead to changes in the colonization status.

To illustrate the contact-mediation processes, we considered an example of a direct contact event where a HCW touched the uncolonized patient (Additional file 2: Table S2). For each touch, a quantity of MRSA was transferred from the HCW’s hand to the patient (HCW*150/2000*ρ_sk_) as well as from the patient to the HCW’s hand (PT_u_*150/2000*ρ_sk_). The transferred MRSA quantities depend on the following: (1) the bacterial concentrations at both contacting surfaces (i.e., HCW and PT_u_), (2) the contact surface area (i.e., 150 sq.cm.), (3) the total surface area (i.e., 2000 sq.cm.), and (4) transfer efficiency. We assumed a symmetrical transfer efficiency, which implied that the fraction of pathogen that was transferred from the HCW to the uncolonized patient was the same as the fraction transferred from the uncolonized patient to the HCW. This fraction is the transfer efficiency of hands to skin or skin to hands (ρ_sk_). The net quantity of pathogens transferred depends on the contamination levels on contacting surfaces. In this case, HCWs were the only sources of MRSA in the uncolonized patient’s room. Thus, the direct contact of HCWs and the uncolonized patient resulted in an increase in contamination of the uncolonized patient, i.e., direct exposure to the uncolonized patient.

Indirect contact involved the contamination of surfaces. When a surface was touched, the contamination from the fingers was assumed to be spread out equally throughout the surface, i.e., the surface was considered spatially homogeneous.

### Model interventions

Our analysis focused on the effect of two environmental cleaning interventions: surface decontamination and surface wiping after touching. Surface decontamination was performed on the entire areas of both porous and nonporous surfaces, once every 24 h. Following each decontamination event, a fraction of MRSA was removed depending on the cleaning efficacy of surface decontamination (ϵ_d_).

Surface wiping was only performed on nonporous surfaces, merely on a fraction of the surface corresponding to the size of the HCW’s hand. After touching a nonporous surface, the HCWs wiped the surface. Thus, the wiping rate (ω_np_) was identical to the rate at which HCWs touched the nonporous surfaces (τ_hcw-sf_). Following each wipe, a fraction of MRSA was removed depending on the cleaning efficacy of wiping (ϵ_w_). Because of the homogeneous nature of the surface, the HCWs wiped a random part of the surface, not necessarily where touching occurred.

### Model analyses

Our analysis is divided into two parts. First, we describe the MRSA contamination levels on all surfaces and skin in the absence of any intervention. We compare the relative importance of direct versus indirect exposure to the HCWs and the uncolonized patient. Direct exposure to the HCW was quantified by the net flow of MRSA resulting from the skin-to-skin contact with the colonized patient, while indirect exposure to the HCW was quantified by the net flow of MRSA from touching the two surfaces in the room. Analogous procedures were used to quantify direct and indirect exposure to the uncolonized patient.

Second, we evaluate and compare the effect of the two interventions: surface decontamination and surface wiping. Surface decontamination and wiping were both evaluated with 50% and 100% cleaning efficacy.

### Sensitivity analyses

We explored the sensitivity of our findings with respect to varying values of the transfer efficiency, die-off rates, and contact rates. The results, presented in the Additional file 3, suggest that our conclusions are robust to parameter uncertainty and variability.

## Results

**Figure 2:**
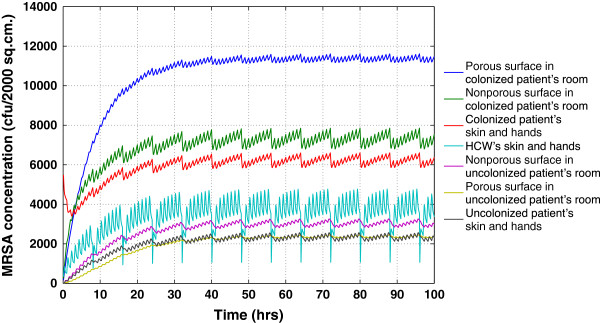
**MRSA concentrations at baseline scenario without intervention.** The Y-axis represents MRSA concentrations on the entire surface area (cfu/2000 cm^2^) from the seven compartments. These compartments starting with the highest MRSA concentrations, are: 1) the porous surface in the colonized patient’s room; 2) the nonporous surface in the colonized patient’s room; 3) the exposed skin and hands of the colonized patient; 4) the exposed skin and hands of the HCW; 5) the nonporous surface in the uncolonized patient’s room; 6) the porous surface in the uncolonized patient’s room; and 7) the exposed skin and hands of the uncolonized patient.

The dynamics of each compartment are shown in Figure 2. MRSA, both on surfaces and skin of patients, quickly came to a steady state. Based on the parameter values shown in Table 1, steady state was reached following a perturbation in approximately 24 h. The relative steady state values can be explained by the fact that the colonized patient was the only source of MRSA; higher MRSA levels found in the room occupied by the colonized patient. Due to the fact that surfaces have slower MRSA die-off rates than the skin, we found higher MRSA levels on surfaces compared to skin. These higher contamination levels on the surfaces reflect the importance of the indirect route (patient to surfaces to HCW) in the colonized patient’s room. In our simulations, 70% of the contamination of the HCW, while in the colonized patient’s room, came through this indirect exposure route. Of this, 66% resulted from touching the nonporous surface rather than the porous surface. We found the situation in the uncolonized patient's room was just reversed; 65% of the contamination of the uncolonized patient came through direct exposure, when the HCW touched the patient.

The relative levels of MRSA contamination on each surface type were reversed in each room (Figure 2). In the colonized patient’s room, the porous surface had higher MRSA contamination, while in the uncolonized patient’s room, the nonporous surface had higher MRSA contamination. This difference occurred despite the equal distribution of the aerosolized shedding on both the porous and nonporous surfaces in the colonized patient’s room. This was because the nonporous surface had a higher transfer efficiency; therefore, transferred more MRSA to the HCW when touched. Thus, the nonporous fomite was more important in facilitating the spread of MRSA, despite having a lower steady-state MRSA contamination level. The porous surface, with the higher MRSA levels, can be thought of as a sentinel fomite. In the uncolonized patient’s room, with no host shedding, the nonporous surface received more contamination due to its higher transfer efficiency; thus, higher MRSA contamination levels would be expected on the nonporous surface compared to the porous surface.

### Surface decontamination

**Figure 3:**
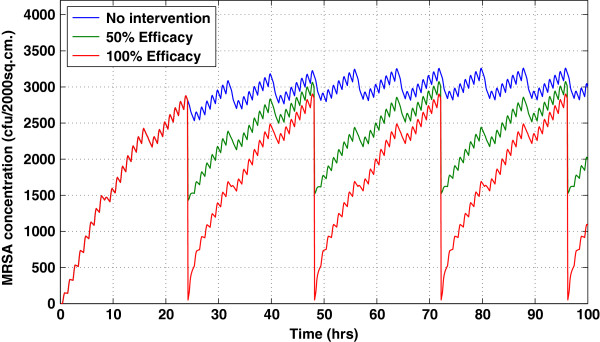
**Temporal effects of daily surface decontamination on the nonporous surface in the uncolonized patient’s room using 50% and 100% efficacy compared to when there is no intervention.** Surface decontamination was performed on both porous and nonporous surfaces. Due to the continuous shedding of the colonized patient, the effect of daily decontamination was short-lived; this pattern is consistently observed in other compartments.

Following decontamination on all surfaces, there was immediate recontamination. Within 24 h, MRSA contamination returned to the pre-decontamination levels (Figure 3). Frequent surface decontamination, therefore, resulted in a more sustained decrease in MRSA contamination.

**Figure 4:**
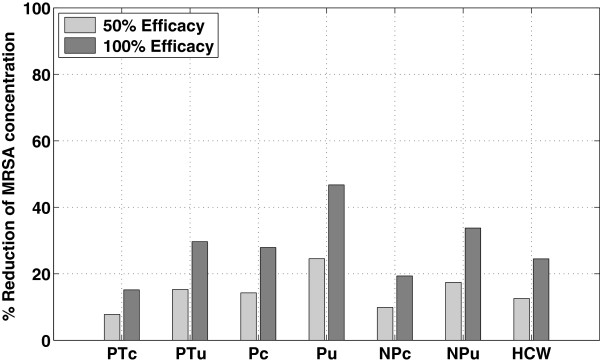
**MRSA reduction in seven compartments due to daily surface decontamination.** The Y-axis is the percentage reduction of MRSA concentration using 50% and 100% efficacy compared to when there is no intervention. The seven compartments are the exposed skin and hands of the colonized patient (PTc), the exposed skin and hands of the uncolonized patient (PTu), the porous surface in the colonized patient’s room (Pc), the porous surface in the uncolonized patient’s room (Pu), the nonporous surface in the colonized patient’s room (NPc), the nonporous surface in the uncolonized patient’s room (NPu), and the exposed skin and hands of the HCW. The porous surface in the uncolonized patient’s room had the highest reduction (46% for 100% efficacy).

These effects trickled throughout the system, as evidenced by the decreased MRSA concentrations on the skin of the colonized patient, uncolonized patient, and HCW, despite not being cleaned directly. With 50% cleaning efficacy, the total exposure to the uncolonized patient was reduced by 15%, while with 100% cleaning efficacy, the exposure was reduced by 30% (Figure 4).

### Surface wiping

**Figure 5:**
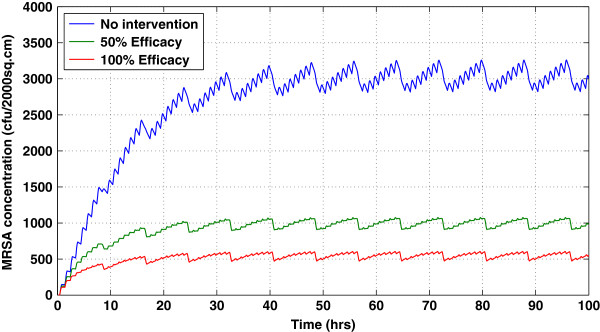
**Temporal effects of surface wiping on the nonporous surface in the uncolonized patient’s room using 50% and 100% efficacy compared to when there is no intervention.** Surface wiping only occurs on nonporous surfaces. Surface wiping consistently suppresses MRSA concentration levels; small fluctuations are due to HCW’s visit to patient’s room every 8-hour.

**Figure 6:**
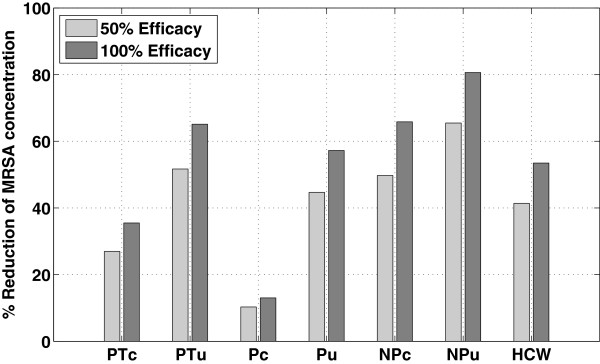
**MRSA reduction in seven compartments due to surface wiping.** The Y-axis is the percentage reduction of MRSA concentration from surface wiping using 50% and 100% efficacy compared to when there is no intervention. The seven compartments are the exposed skin and hands of the colonized patient (PTc), the exposed skin and hands of the uncolonized patient (PTu), the porous surface in the colonized patient’s room (Pc), the porous surface in the uncolonized patient’s room (Pu), the nonporous surface in the colonized patient’s room (NPc), the nonporous surface in the uncolonized patient’s room (NPu), and the exposed skin and hands of the HCW. The nonporous surface in the uncolonized patient’s room had the highest reduction (81% for 100% efficacy).

With surface wiping, there was no jagged pattern, as seen with daily decontamination; instead, there was a cyclical pattern due to the HCW visits to patient rooms (Figure 5). Surface wiping with 50% cleaning efficacy reduced the total exposure to the uncolonized patient substantially (52% reduction in exposure), while with 100% cleaning efficacy, the increased effect was less than linear (65% reduction in exposure). Because surface wiping was effectively targeted as intervention against MRSA on nonporous surfaces, the nonporous surfaces were affected more by wiping than porous surfaces; in particular, the nonporous surface in the uncolonized patient’s room had the highest reduction (81%, given a 100% cleaning efficacy) (Figure 6).

### Comparison of surface decontamination and surface wiping

The efficacy of surface decontamination and surface wiping are both depend on the following factors: (1) total surface area cleaned per day, (2) cleaning frequency, (3) proportion of surface area cleaned at each implementation, (4) type of surface cleaned, (5) cleaning efficacy, and (6) time of cleaning. Surface decontamination was assumed to clean the whole surface one to three times daily, whereas surface wiping cleaned a random proportion of the surface (150 cm^2^/2000 cm^2^) eight times per hour. To compare the two intervention approaches, cleaning efficacy was held constant, at 100%. Surface decontamination always starts at time zero, and HCWs wiped after every touch, unless specified differently. Following surface touching, MRSA contamination was spread out equally throughout the surface. We made a similar assumption that the HCW wiped a random portion of the surface, not necessarily where touching occurred.

We varied the total surface area cleaned per day by varying the cleaning frequency and surface area cleaned at each implementation (Table 2). Daily surface decontamination affected a large surface area at each implementation (four surfaces, each with 2000 cm^2^), while surface wiping affected a much smaller surface area (150 cm^2^) with each wipe. However, because the cleaning frequency of wiping was many times per hour, wiping cleaned a much larger total surface area per day (57600 cm^2^/day vs. 8000 cm^2^/day).

**Table 2:** Intervention effectiveness when total surface area cleaned per day is varied

**Cleaning frequency**	**No**	**Surface decontamination**			**Surface wiping**	
	**Intervention**	**A. Every 24 h**	**B. Every 12 h**	**C. Every 8 h**	**D. HCW swipe 8 times/h**	E. HCW swipe 3 times/h*
Total surface area cleaned/day (cm^2^)		8000	16000	24000	57600	24000
Types of surfaces		Both	Both	Both	Nonporous	Nonporous
Number of cleaning events/day		1	2	3	192	80
Surface area cleaned each implementation		2000	2000	2000	150	150
Average MRSA concentration on the uncolonized patient	2065.55	1454.76	1055.09	776.87	703.89	1111.86
Percent reduction of exposure to the uncolonized patient	n/a	29.57%	48.92%	62.38%	65.92%	46.17%

*HCWs touch the nonporous surface 8 times an hour and wipe only 3 times an hour.

When surface decontamination was performed daily (Table 2 scenario A) and surface wiping was performed after each surface touch by HCWs (Table 2 scenario D), we found wiping was more effective. When surface decontamination was performed once every eight hours (Table 2 scenario C), the uncolonized patient had similar levels of MRSA exposure, despite the fact that surface-decontamination cleaned less than half the surface area that surface wiping could clean. We found that the surface decontamination performed better than surface wiping, when the two interventions were adjusted to clean the same total daily surface area (Table 2 scenarios C and E). However, this was an artifact that the HCW did not clean the specific area that they contaminated. To address this, incorporating spatial heterogeneity in the modeling of the surfaces is required.

## Discussion

Our model for MRSA exposure assessment highlights the dynamic interplay between MRSA colonized and uncolonized patients, HCWs, and environmental surfaces. Environmental cleaning has been under-appreciated; therefore, underutilized as an intervention option in the hospital setting. The analysis presented here highlights the importance of the indirect exposure route, which suggests the potential of environmental cleaning of surfaces as an integral component of MRSA infection control.

Specifically, our analysis suggests that environmental surfaces are key exposure sources that contaminate the hands. A randomized crossover study on the impact of enhanced cleaning in intensive care units (ICUs) supports our findings that enhanced cleaning of high-contact surfaces not only significantly reduced the number of MRSA isolates in the environment but also on the hands of the staff [32]. These conclusions remained, even when HCWs touched patients as often as they touched surfaces, largely due to the higher die-off rates on hands and skin compared to surfaces. In reality, HCWs touch room surfaces more frequently than they touch patients [33], indicating that we might be underestimating the importance of indirect exposure through environmental surfaces.

HCWs are frequently viewed as vectors of transmission, also assumed in our model [17,19]. A study of an isolation ward for six epidemic MRSA (EMRSA) patients provides strong evidence that the transient and short-term carriage in HCWs is not uncommon and probably results in the transfer of EMRSA between patients [34]. However, HCWs may also be the source of transmission [17]. A six-year surveillance of an operating room technician, who was colonized with *S. aureus*, demonstrated transmission from this technician to patients in a number of situations [35]. Since our model only considered the HCWs as mechanical vectors, we probably underestimated the exposure to the uncolonized patient.

Given the role of HCW’s hands in spreading contamination, hand hygiene is potentially a strong effect modifier of cleaning. This is clearly true if hand hygiene is done perfectly, i.e., if contamination never gets on the hand, surface cleaning is irrelevant. In reality, however, hand hygiene is never perfectly implemented. Real-world impediments include the following: (1) imperfect compliance with hand hygiene procedures [36], (2) cross-contamination of HCW hands when HCWs touch their own contaminated clothing [16,18], and (3) recontamination due to the touching of contaminated surfaces [15]. Because of these complications, we chose not to include hand hygiene in this analysis as it would have required a more sophisticated model, which would incorporate spatial heterogeneity of the skin, i.e., differentiating between the hand and other parts of the body. Although beyond the scope of this manuscript, describing the joint effects of hand hygiene (considering compliance and recontamination) and surface cleaning would provide valuable information for developing realistic intervention strategies.

Because of these limitations, implementing a hand hygiene intervention in conjunction with other interventions is necessary to decrease MRSA transfer and exposure to the uncolonized patients. Surface decontamination cleans thoroughly entire surfaces, when implemented, but it cannot feasibly do so very often. Surface wiping is less thorough, only cleans very small segments of surfaces, but does so quite often. Despite the lack of thoroughness, we found that surface wiping was more effective than daily surface decontamination as the former cleaned more surface area per day. Furthermore, our assumption that surfaces were homogeneous underestimated the importance of wiping, where wiping generally cleaned areas that had been touched rather than cleaning the surface proportionally, in an untargeted manner.

While new technology that provides high levels of disinfection (e.g., hydrogen peroxide vapor) has been shown to effectively eliminate bacteria from the environment [37,38], our analysis showed diminishing returns with increasing cleaning efficacy due to recontamination. Consistent with our finding, a prior study demonstrated that after eliminating MRSA from the environment by applying hydrogen peroxide vapor, recontamination occurred 24 hours after colonized patients were readmitted into the intensive care unit [37]. Further simulations suggested that by increasing the frequency of cleaning, additional reductions in the exposure to the uncolonized patient were achievable; i.e., increasing cleaning frequency was more important than increasing cleaning efficacy.

Our model assumed that surface decontamination thoroughly cleaned the entire surface. In reality, such thoroughness is often lacking. A review of environmental hygiene in healthcare settings showed that implementation often lacked thoroughness; only 40% of the surfaces were being thoroughly cleaned in accordance with existing policies [39]. Some have used a monitoring system to improve cleaning thoroughness, which resulted in decreased MRSA infection [40,41]. However, this approach often requires an extra person; for example, when hand-touched-surfaces near patients and HCWs’ station were cleaned more frequently by extra personnel, there was a 32.5% reduction in environmental contamination sites and 26.6% reduction in new MRSA infections [41].

Similarly, our model assumed that surface wiping was conducted using proper techniques. The true effectiveness of surface wiping was difficult to define due to a wide variety of commercially available wipes and microfiber-based fabrics, as well as a variation in test protocols [21,42]–[47]. One study showed that wiping plastic surfaces three or more times with saline-moistened wipes is as effective as disinfectant wipes [45]. At the same time, disinfectant wipes may serve as vectors in transferring pathogen between surfaces when reused without proper cleaning [46,47]. Nevertheless, as suggested in our analysis, the frequency of wiping with presumably good technique can significantly reduce the environmental contamination.

In addition, our findings are helpful in interpreting environmental surveillance data. When a MRSA-positive patient has recently been in a hospital room, our model will predict a higher concentration of MRSA on porous surfaces (e.g., bed sheets, mattresses, and blankets), all else being equal between the surfaces. On the other hand, for a MRSA-negative patient, our model will predict a higher concentration of MRSA on nonporous surfaces. This suggests the possibility that porous surfaces can act as a sentinel fomite indicating the recent presence of a MRSA-positive patient. Further analysis and data are required to support this finding.

## Conclusions

In this study, we modeled only fate and transport, making no prediction on infection risk or any other interpretation of the total exposure dose to the uncolonized patient. According to the quantitative risk assessment paradigm, exposure assessment is an initial and essential step toward improving our understanding in transmission system. To develop a full environmental infection transmission system, a model requires a dose–response function in the mass action formulation. Our dynamic fate and transport modeling framework highlights that developing optimal control strategies depends on a number of environmental factors such as pathogen die-off rates on surfaces and hands, the transfer efficiency of pathogen movement from hand to surface and surface to hand, and a host of behavioral features that include rates of touching surfaces as well as colonization sites such as the nose and the skin. Improved data on these processes along with more detailed models that contain site-specific features would provide guidance on not just the effectiveness of any particular control strategy, but also the joint effects of multiple intervention strategies.

## Competing interests

The authors declare that they have no competing interests.

## Authors’ contributions

NP designed the study, constructed the model, performed the analysis, and drafted the manuscript. IHS, JSK, and JNSE reviewed the model, and provided critical advice. NP wrote the manuscript with major contributions from JNSE and other authors. All authors have read and approved the final manuscript.

## Pre-publication history

The pre-publication history for this paper can be accessed here:

http://www.biomedcentral.com/1471-2334/13/595/prepub

## Supplementary Material

Additional file 1: Supplementary material I Supporting Evidence for Environment-mediated Transmission and Model Parameterization. Click here for file

Additional file 2: Supplementary material II Table S2 and differential equations. Click here for file

Additional file 3: Supplementary material III Sensitivity analysis. Click here for file
