# Supplementary material for: The dynamics of methicillin-resistant Staphylococcus aureus exposure in a hospital model and the potential for environmental intervention
Source: BMC Infect Dis. 2013 Dec 17;13:595. doi: 10.1186/1471-2334-13-595 (PMC3878576; doi:10.1186/1471-2334-13-595)
Supplement: Additional file 1: Supplementary material I — Supporting Evidence for Environment-mediated Transmission and Model Parameterization. [file 1471-2334-13-595-S1.docx]

Supplemental Material I

Supporting Evidence for Environment-mediated Transmission and

Model Parameterization

To perform exposure assessment in a hospital environment, we used an environmental infection transmission system (EITS) framework that allows the incorporation of the pathogen, environment, patients, and healthcare workers (HCWs) in the same system. Here, we provide supporting evidence for the parameterization of the following environmental processes: (1) *Staphylococcus aureus* (*S. aureus*) shed into the environment; (2) *S. aureus* survivability in the environment and on hands; (3) contact between hands and surfaces and between the fingertip and nose; and (4) *S. aureus* transferred between the two contacting surfaces (porous and nonporous).

S1. *S. aureus* was shed into the environment continuously and sometimes profusely

*S. aureus* is a major human pathogen that commonly colonizes individuals without causing pathology. Its ecological niches are the anterior nares, the skin particularly the perineum, and the throat [[1](#_ENREF_1)]. Other carriage sites may also include axillae, hands, forearms, and gastrointestinal tract. *S. aureus* can be found ubiquitously in hospital environment on various surfaces, including floors, carpets, bed linens, bed frames, over-bed tables, blood pressure cuffs, nurse call buttons, as well as on nurse stations and furniture in public areas [[2](#_ENREF_2), [3](#_ENREF_3)]. The role of fomites and air in exposure is complex; important studies in the late 1950s and early 1960s attempted to address this issue.

Experimental studies in 1956 and 1958 suggested that the expulsion of *S. aureus* as droplets or droplet nuclei from the nose and mouth into the air was quantitatively of much less importance compared to indirect pathways involving fomites [1, 2]. These indirect pathways include the egress of *S. aureus* from nasal secretions that contaminate skin, clothing, and bedding. These contamination sites could then release *S. aureus* into the free air by friction or movement.

By the early 1960s, much attention was focused on the ability of *S. aureus* to disperse into air, but the underlying mechanism was unclear. There were questions regarding whether *S. aureus* could float freely in the air or attach to textile fibers [[4](#_ENREF_4)]. Desquamated epithelial cells were found in the air as early as 1855, and the possibility that they could carry organisms was suggested in 1905. However, not until 1962 was it discovered that these cells carry most of the skin organisms dispersed into the air in hospitals [[4](#_ENREF_4), [5](#_ENREF_5)].

The average human skin surface area is 1.75 m^2^. This surface area comprises approximately 2 × 10^9^ epithelial cells. A complete layer of cells can be lost and replaced every 24 hours on average. Hence, at least 10^7^ cells may be shed every day [[6](#_ENREF_6), [7](#_ENREF_7)]. It has been estimated that each airborne desquamated epithelial cell could carry four viable cocci of *S. aureus* [[8](#_ENREF_8)].

Several factors influence dispersal heterogeneity. First, the location of colonization can affect the dispersal quantity. Perineal carriers tend to be heavy dispersers [[9-11](#_ENREF_9)], while some nasal carriers do not disperse at all. In one study, 62% of 87 nasal carriers dispersed *S. aureus* into the air (13). Second, the skin conditions can also affect the dispersal quantity. Patients with dermatitis, psoriasis, or those with open wounds can be heavy dispersers [[12](#_ENREF_12)]. Other factors that may increase dispersal are movement, clothing, hand washing, showering, and bathing [[12-14](#_ENREF_12)]. On the other hand, receiving decolonization or systemic antibiotic treatment for *S. aureus* can decrease or prevent the dispersal into air [[15](#_ENREF_15)]. In addition to these environmental and host factors, there also exists dispersal variability within the same individual [[11](#_ENREF_11), [16](#_ENREF_16)].

S1.1 Shedding

Reported ranges in the amount of *S. aureus* dispersed are summarized in Table S1. Airborne epithelial cells range in sizes from 4 to 20 μm, with a median diameter of 14 (13–17) μm [[17](#_ENREF_17)]. We assumed that the number of particles contained in 1 ft^3^ of air is approximately equal to the number that settle on 1 ft^2^ in 1 min, which is valid for the particle range we are interested in [[18](#_ENREF_18)]. Therefore, in Table S1, the reported *S. aureus* air count is expressed as cfu/cm^2^/min for use in the model, assuming that all *S. aureus* in the air completely settle on the horizontal surfaces. In our model, the value used for the shedding parameter was 1 × 10^-2^ cfu/cm^2^/min, which is within the range of the experimental studies.

**Table S1**. Summary of source literature for shedding parameter

| **Author/Year** | **Study Design** | **Subject** | **Setting** | **Method** | ***S. aureus* count (cfu/cm^2^/min^a^)** |
| --- | --- | --- | --- | --- | --- |
| Hare, 1956 [[19](#_ENREF_19)] | Experimental study: Results in this table are from the 5^th^ experiment | 3 *S. aureus* nasal carriers and 2 non-carriers | Subjects were fully clothed and exercised for 15 minutes. | Settling plates in a cubicle | 0.14–47.4 cfu/ft^2^/min  (1.5 × 10^-4^–5 × 10^-2^ cfu/cm^2^/min) |
| Hare, 1958 [[10](#_ENREF_10)] | Experimental study | 19 nasal carriers and 7 non-carriers | Each day, subjects wore clothing and exercised in the cubicle. | Settling plates in a cubicle | 0–27.8 cfu/ft^2^/min  (0–3 × 10^-2^ cfu/cm^2^/min) |
| Noble, 1965 [[6](#_ENREF_6)] | Experimental study | 127 subjects (staff, students, hospital in-patients, patients with skin diseases) | Subjects undressed in a cubicle during a 2-minute period | Air sampling through slit samplers of a cubicle | 0.25–100 cfu/ft^3^/2 min  (1.3 × 10^-4^–5 × 10^-2^ cfu/cm^2^/min) |
| Noble, 1962 [[20](#_ENREF_20)] | 4-year environmental surveillance study in 3 male surgical wards | 3,675 patients (1488 were *S. aureus* nasal carriers on admission) | Various ward activities | Air sampling through slit samplers for 2 h in wards | 0–3 cfu/ft^3^/min  (0–3.2 × 10^-3^ cfu/cm^2^/min) |
| Williams, 1967 [[18](#_ENREF_18)] | 20-month surveillance study | 307 patients were admitted. | Various ward activities | Settling plates in the rooms and corners of the ward | 0–700 cfu/ft^2^/h  (0–1.3 × 10^-2^ cfu/cm^2^/min) |
| Hill, 1974 [[21](#_ENREF_21)] | Experimental study | 615 laboratory technicians, doctors, and nurses: 238 males & 377 females. | Subjects moved arms and legs in a defined manner at a constant rate. | Air sampling in a test chamber for 2 min | 0–2800 cfu/100 ft^3^/2 min  (0–1.5 × 10^-2^ cfu/cm^2^/min) |
| Gehanno, 2009 [[15](#_ENREF_15)] | Environmental sampling study in hospital wards | 24 patients infected or colonized with MRSA. | Patients were in their beds with no movement. | Air sampling for 10 min, which represents 1 m^3^ | 1–78 cfu/m^3^/10 min  (1 × 10^-5^–7.8 × 10^-4^ cfu/cm^2^/min) |

a) We assumed that the number of microorganisms contained in 1 ft^3^ of air is equal to the number that settle on 1 ft^2^ in 1 minute [[18](#_ENREF_18)]. One ft is 30.5 cm. One ft^2^ is 930.25 cm^2^.

S2. *S. aureus* survives and remains viable on surfaces and hands for a long period of time

*S. aureus* is known to survive in a variety of environmental niches by virtue of its adaptability and resistance to environmental stress [[22](#_ENREF_22), [23](#_ENREF_23)]. Studies have shown that strains causing epidemics associated with environmental contamination had longer survival than non-epidemic strains [[23-25](#_ENREF_23)]. Some staphylococcal epidemic strains may persist on surfaces for months [[23](#_ENREF_23), [25-27](#_ENREF_25)]. The prolonged survivability of *S. aureus* in the environment not only contributes to its ability to disseminate but also makes decontamination in the hospital environment both more difficult and more important.

S2.1 Survival parameter

Many studies have been performed to investigate the survival of various nosocomial pathogens in hospital, household, or experimental settings [[23](#_ENREF_23), [24](#_ENREF_24), [28-33](#_ENREF_28)]. However, study designs, study conditions, and the outcome measures were not all consistent. Measures used included death rate per unit time [[23](#_ENREF_23), [24](#_ENREF_24), [28](#_ENREF_28), [29](#_ENREF_29)], amount changes or percent (%) recovery over time [[30-32](#_ENREF_30)], and survival time in days [[33](#_ENREF_33)]. We selected references with quantitative measures that allow calculation of the die-off rate (μ) based on the initial and final concentrations over time, as shown in Equation S1 [[29](#_ENREF_29)].

$$\mu= \frac{{log}_{10}(M_{0})-{log}_{10}(M_{t})}{T_{survival}}$$

where, *M_t_* is $M_{o}*{10}^{-\mu t}$ (S1)

S2.2 Survival on porous surfaces

A study was performed to evaluate *S. aureus* survival on contaminated standardized sterile fabrics commonly used in dental clinics [[34](#_ENREF_34)]. These results suggested that *S. aureus* could survive for 3–7 days on surfaces, including cotton/polyester fabric and paper. Based on these data, we estimated the die-off rate for cotton/polyester fabric as 0.000632 log cfu/min and used it as porous surface die-off rate in our model.

S2.3 Survival on nonporous surfaces

Laboratory experiments on decay rates of six different nonporous surfaces found a much higher level of inactivation by using the culture method in comparison to the quantitative PCR method [[29](#_ENREF_29)]. Based on this study, we derived the decay rate by the culture method on plastic and measured it to be 0.012 log cfu/h (0.0002 log cfu/min).

S2.4 Survival on hands and skin

One study that involved the application of *S. aureus* onto the fingertips of four volunteers found that the greatest pathogen loss occurred in the first five minutes [[31](#_ENREF_31)].The author concluded that the loss was due to desiccation. After the initial five minutes, the decline was less pronounced. In this study, we used the data of this second phase (after the initial five minutes), assuming a first-order decay. The die-off rate on the fingertip was estimated as 0.00353 log cfu/min in the study mentioned above, and the same was used in our model.

Despite its ability to colonize on the skin, *S. aureus* survives for shorter period of time on hands than on surfaces. This characteristic is not unique to *S. aureus*; other nosocomial pathogens such as *Candida* species, enterococci, and *Klebsiella* species, also have shorter survival on hands than on surfaces [[35-37](#_ENREF_35)].

**S3. *S. aureus* picked up by hands and sometimes deposited to nose**

A study was conducted in a 12-bed UK adult general ICU to measure the contact rate by healthcare workers with patients (direct contact) and with the patients’ immediate environment (indirect contact) [[38](#_ENREF_38)]. This study reported that on average, each patient was contacted directly 159 (95% confidence intervals (CI) 144–178) and indirectly 191 (95% CI 174–121) times/day. Notably, there were more indirect contacts compared to direct contacts. In our model, the HCW visits each patient’s room for 20 minutes per hour. During this time, the HCW touches the patient with the same rate that the HCW touches the porous and nonporous surfaces, i.e., 8 times per hour. While this rate represents contact rates reported in the above study, contact rates in different institutions or different clinical settings may vary.

The rate at which a fingertip touches the nasal and conjunctival mucosa was examined in a study on rhinovirus transmission, where medical and non-medical personnel seated in a conference room were observed [[39](#_ENREF_39)]. The average reported rate was, on average, 0.33 times per person-hour of observation (i.e., 0.005 times per minute). Another study that examined the frequency at which adults touched their nostrils reported a frequency range of 1 to 30 times within a continuous 3 h of observation (i.e., 0.0055 to 0.167 times per minute) [[40](#_ENREF_40)]. In this model, we chose the rate of 0.025 times per minute, the mid-range of these studies.

S4. *S. aureus* transferred between contacting surfaces.

There are three types of contacts, i.e., direct contact of the hand and skin, indirect contact of the hand and surface, and contact of the fingertip and the nose. Following each contact, pathogens can be transferred between the two contacting surfaces. Several factors can influence the transfer between surfaces: the nature of the environmental surfaces, moisture, temperature, relative humidity in the air, pressure applied during contact, amount of bacteria on both the contacting surfaces, as well as the bacterial species [[41](#_ENREF_41), [42](#_ENREF_42)].

S4.1 Transfer efficiency

Transfer efficiency is a measure of the fraction of the organisms on one surface that is transferred to another contacting surface. In general, surfaces are referred to as one of the following categories: porous and nonporous or textile and non-textile. We used the former category in this study. Despite a wide range of gross characteristics, a porous surface was referred to as a surface with pores or deep recesses where organisms may reside. Conversely, a nonporous surface was frequently a hard and smooth surface that does not offer crevices in which microorganisms may hide.

S4.2 Transfer from hands to surfaces and from surfaces to hands

A study reported in 1990 investigated the survival and transfer of 5 different organisms including *S. aureus* [[32](#_ENREF_32)]. The laminate surfaces were contaminated, and the amount transferred to hands was measured at 0, 1, 2, and 24 h after contamination. Organisms were transferred more efficiently from laminate surfaces than from clothes. The transfer efficiency from laminate surfaces to hands was the highest at one hour after contamination (43%) and decreased subsequently. Similarly, the transfer efficiency from contaminated clothes to hands was also the highest at one hour after contamination (5%). At 24 h, however, there appeared to be regrowth of *S. aureus*. This led to higher measures in transfer efficiency, which were concluded to be spurious.

The transfer efficiency of *S. aureus* from fabrics (100% cotton and 50–50% cotton-polyester) to the finger pads of adult volunteers were tested using moist, dry, and re-moistened pieces of fabrics, with or without friction during contact [[41](#_ENREF_41)]. Higher levels of transfer occurred between moist donors and/or recipients surfaces, when friction was applied. Transfer efficiency was reported to be within a range of <0.1 to 2.5% cfu, depending on the environmental condition. Another study examined the transfer efficiency of microorganisms from surfaces to hands and from the fingertip to the lower lip, by using a different protocol than that previously described [[42](#_ENREF_42)]. The study found a significant difference in the transfer efficiency between porous and nonporous surfaces. The transfer efficiency for nonporous surfaces ranged from 28 to 66%, while that for porous surfaces was mostly <1%.

Despite having different experimental designs and measurement methods, all the above mentioned studies indicated that nonporous surfaces have a higher transfer efficiency than porous surfaces. Because of the concern that the environmental exposure of pathogens is a human risk, the transfer efficiency has generally been measured from surfaces to humans and not from humans to surfaces. In reality, however, it is possible that each contact results in a bidirectional pathogen flow between the contacting surfaces. In this model, we assumed symmetrical transfer efficiency. We chose the transfer efficiency values of 0.4 and 0.1 for nonporous and porous surfaces, respectively.

S4.3 Transfer from fingertip to nose and from nose to fingertip

To the best of our knowledge, there have been no studies on the transfer efficiency from hand to nose. However, bacterial transfer efficiency from the fingertip to the lower lip was measured to be within a range of 34 to 41% [[42](#_ENREF_42)]. In our model, we assumed that the transfer efficiency from hand to nose is much less than that from the fingertip to the lip, given that there is less direct contact of the fingertip to the anterior nares, where *S. aureus* resides. We considered 0.2 as the transfer efficiency for the fingertip to the nose.

S4.4 Transfer from hand to hand

There are no studies on the transfer efficiency from hand to hand. We assumed that the efficiency of transfer from hand to hand would be similar to that from the fingertip to the lip [[42](#_ENREF_42)].

**Reference**

1. Verbrugh HA: **Colonization with *Staphylococcus aureus* and the role of colonization in causing infection.** In: *Staphylocci in human disease.* 2nd edn. Edited by Crossley KB, Jefferson,K.K., Archer,G.L., Fowler Jr,V.G.: Wiley-Blackwell; 2009: 255-271.

2. Cimolai N: **MRSA and the environment: implications for comprehensive control measures**. *Eur J Clin Microbiol Infect Dis* 2008, **27**(7):481-493.

3. Dancer SJ: **Importance of the environment in meticillin-resistant *Staphylococcus aureus* acquisition: the case for hospital cleaning**. *Lancet Infect Dis* 2008, **8**(2):101-113.

4. Davies RR, Noble WC: **Dispersal of bacteria on desquamated skin**. *Lancet* 1962, **2**(7269):1295-1297.

5. Davies RR, Noble WC: **Dispersal of staphylococci on desquamated skin**. *Lancet* 1963, **1**(7290):1111.

6. Noble WC, Davies RR: **Studies on the Dispersal of Staphylococci**. *J Clin Pathol* 1965, **18**:16-19.

7. Mackintosh CA, Lidwell OM, Towers AG, Marples RR: **The dimensions of skin fragments dispersed into the air during activity**. *J Hyg* 1978, **81**(3):471-479.

8. Lidwell OM, Noble WC, Dolphin GW: **The use of radiation to estimate the numbers of micro-organisms in airborne particles**. *J Hyg* 1959, **57**:299-308.

9. Boe J, Solberg CO, Vogelsang TM, Wormnes A: **Perineal Carriers of Staphylococci**. *Br Med J* 1964, **2**(5404):280-281.

10. Hare R, Ridley, M.: **Further studies on the transmission of Staph. aureus.** *Br Med J* 1958:1(Suppl 5062):69-73.

11. Solberg C: **A study of carriers of *Staphylococcus aureus* with special regard to quantitative bacterial estimations**. *Acta Med Scand* 1965, **436**:1-96.

12. Williams RE: **Epidemiology of airborne staphylococcal infection**. *Bacteriol Rev* 1966, **30**(3):660-674.

13. Meers PD, Yeo GA: **Shedding of bacteria and skin squames after handwashing**. *J Hyg* 1978, **81**(1):99-105.

14. Plano LR, Garza AC, Shibata T, Elmir SM, Kish J, Sinigalliano CD, Gidley ML, Miller G, Withum K, Fleming LE*,* Solo-Gabriele HM: **Shedding of *Staphylococcus aureus* and methicillin-resistant *Staphylococcus aureus* from adult and pediatric bathers in marine waters**. *BMC Microbiol* 2011, **11**(1):5.

15. Gehanno JF, Louvel A, Nouvellon M, Caillard JF, Pestel-Caron M: **Aerial dispersal of meticillin-resistant *Staphylococcus aureus* in hospital rooms by infected or colonised patients**. *J Hosp Infect* 2009, **71**(3):256-262.

16. Solberg CO: **Spread of *Staphylococcus aureus* in hospitals: causes and prevention**. *Scand J Infect Dis* 2000, **32**(6):587-595.

17. Noble WC, Lidwell OM, Kingston D: **The size distribution of airborne particles carrying micro-organisms**. *J Hyg* 1963, **61**:385-391.

18. Williams RE: **Airborne staphylococci in the surgical ward**. *J Hyg* 1967, **65**(2):207-217.

19. Hare R, Thomas CGA: **The transmission of *Staphylococcus aureus***. *Br Med J* 1956:840-844.

20. Noble WC: **The dispersal of staphylococci in hospital wards**. *J Clin Pathol* 1962, **15**(6):552-558.

21. Hill J, Howell A, Blowers R: **Effect of clothing on dispersal of *Staphylococcus aureus* by males and females**. *Lancet* 1974, **2**(7889):1131-1133.

22. Clements MO, Foster SJ: **Stress resistance in *Staphylococcus aureus***. *Trends Microbiol* 1999, **7**(11):458-462.

23. Farrington M, Brenwald N, Haines D, Walpole E: **Resistance to desiccation and skin fatty acids in outbreak strains of methicillin-resistant *Staphylococcus aureus***. *J Med Microbiol* 1992, **36**(1):56-60.

24. Rountree PM: **The effect of desiccation on the viability of *Staphylococcus aureus***. *J Hyg* 1963, **61**:265-272.

25. Wagenvoort JH, Sluijsmans W, Penders RJ: **Better environmental survival of outbreak vs. sporadic MRSA isolates**. *J Hosp Infect* 2000, **45**(3):231-234.

26. Layton MC, Perez M, Heald P, Patterson JE: **An outbreak of mupirocin-resistant *Staphylococcus aureus* on a dermatology ward associated with an environmental reservoir**. *Infect Control Hosp Epidemiol* 1993, **14**(7):369-375.

27. Rampling A, Wiseman S, Davis L, Hyett AP, Walbridge AN, Payne GC, Cornaby AJ: **Evidence that hospital hygiene is important in the control of methicillin-resistant *Staphylococcus aureus***. *J Hosp Infect* 2001, **49**(2):109-116.

28. Beard-Pegler MA, Stubbs E, Vickery AM: **Observations on the resistance to drying of staphylococcal strains**. *J Med Microbiol* 1988, **26**(4):251-255.

29. Masago Y, Shibata T, Rose JB: **Bacteriophage P22 and *Staphylococcus aureus* attenuation on nonporous fomites as determined by plate assay and quantitative PCR**. *Appl Environ Microbiol* 2008, **74**(18):5838-5840.

30. McDade JJ, Hall LB: **Survival of *Staphylococcus aureus* in the environment. I. Exposure of surfaces**. *Am J Hyg* 1963, **78**:330-337.

31. Gontijo Filho PP, Stumpf M, Cardoso CL: **Survival of gram-negative and gram-positive bacteria artificially applied on the hands**. *J Clin Microbiol* 1985, **21**(4):652-653.

32. Scott E, Bloomfield SF: **The survival and transfer of microbial contamination via cloths, hands and utensils**. *J App Bacteriol* 1990, **68**(3):271-278.

33. Neely AN, Maley MP: **Survival of enterococci and staphylococci on hospital fabrics and plastic**. *J Clin Microbiol* 2000, **38**(2):724-726.

34. Cuesta A, Nastri N, Bernat M, Brusca M, Turcot L, Nastri M, Rosa AC: **Survival of *Staphylococcus aureus* on fomites**. *Acta odontologica latinoamericana : AOL* 2008, **21**(2):141-146.

35. Traore O, Springthorpe VS, Sattar SA: **A quantitative study of the survival of two species of Candida on porous and non-porous environmental surfaces and hands**. *J Appl Microbiol* 2002, **92**(3):549-555.

36. Noskin GA, Stosor V, Cooper I, Peterson LR: **Recovery of vancomycin-resistant enterococci on fingertips and environmental surfaces**. *Infect Cont Hosp Epid* 1995, **16**(10):577-581.

37. Hart CA, Gibson MF, Buckles AM: **Variation in skin and environmental survival of hospital gentamicin-resistant enterobacteria**. *J Hyg* 1981, **87**(2):277-285.

38. McArdle FI, Lee RJ, Gibb AP, Walsh TS: **How much time is needed for hand hygiene in intensive care? A prospective trained observer study of rates of contact between healthcare workers and intensive care patients**. *J Hosp Infect* 2006, **62**(3):304-310.

39. Hendley JO, Wenzel RP, Gwaltney JM, Jr.: **Transmission of rhinovirus colds by self-inoculation**. *N Engl J Med* 1973, **288**(26):1361-1364.

40. Nicas M, Best D: **A study quantifying the hand-to-face contact rate and its potential application to predicting respiratory tract infection**. *J Occup Environ Hyg* 2008, **5**(6):347-352.

41. Sattar SA, Springthorpe S, Mani S, Gallant M, Nair RC, Scott E, Kain J: **Transfer of bacteria from fabrics to hands and other fabrics: development and application of a quantitative method using *Staphylococcus aureus* as a model**. *J Appl Microbiol* 2001, **90**(6):962-970.

42. Rusin P, Maxwell S, Gerba C: **Comparative surface-to-hand and fingertip-to-mouth transfer efficiency of gram-positive bacteria, gram-negative bacteria, and phage**. *J Appl Microbiol* 2002, **93**(4):585-592.
