# Supplementary material for: The dynamics of methicillin-resistant Staphylococcus aureus exposure in a hospital model and the potential for environmental intervention
Source: BMC Infect Dis. 2013 Dec 17;13:595. doi: 10.1186/1471-2334-13-595 (PMC3878576; doi:10.1186/1471-2334-13-595)
Supplement: Additional file 2: Supplementary material II — Table S2 and differential equations. [file 1471-2334-13-595-S2.pdf]

Supplemental Material II

Table S2. Submodel for a direct contact event between a healthcare worker (HCW) and the uncolonized patient (PTu). HCW represents the concentration of MRSA cfu on the HCW (MRSA cfu/2000 sq.cm.). PTu represents the concentration of MRSA cfu on the uncolonized patient (MRSA cfu/2000 sq.cm.). The transfer efficiency of MRSA from the HCW's hands to the uncolonized patient's skin was assumed to be the same as transfer efficiency from the uncolonized patient's skin to the HCWs' hands.

	**Healthcare worker (HCW)**	**Uncolonized patient (PTu)**
Total surface area (sq.cm.)	2000	2000
Contact surface area (sq.cm.)	150	150
Transfer efficiency (ρ_{sk})	0.35	0.35

Bidirectional flows between the two contacting surfaces:

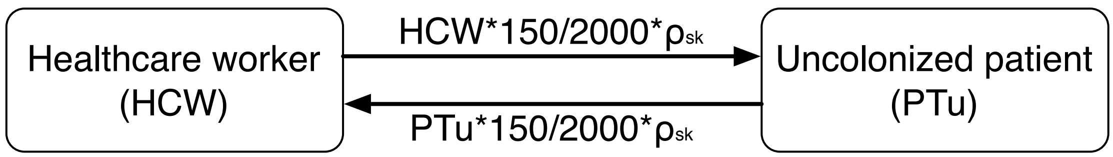

Differential equations

A diagram depicting the compartmental model is shown in Figure 1 in the main text. The definitions of the parameters are presented in Table 1 and also in the main text. The following are the differential equations for the ten compartments.

1) The colonized patient (PT_c)

We assumed that the colonized patient maintains a steady MRSA concentration on the exposed skin and hands (PT_c). This balance was achieved by the gain and loss of MRSA. The colonized patient gained MRSA from the replenishment of the skin epithelial cells and from touching the nose. The replenishing rate was assumed to be the same as the dispersal rate. Concentration of MRSA in the nose (PT_{cn}) was assumed constant (1000 cfu/4 cm^2). The colonized patient, on the other hand, lost MRSA from the natural die-off and from pathogen flows to surfaces and HCWs due to touching events. The HCWs touched the colonized patient only during the first 20 minutes of the hour.

The change in MRSA concentration on the skin and hand of the colonized patient (PT_c) are given by the following:

$$\frac{dPT_c}{dt} = \alpha A_{pt} - PT_c \frac{A_f}{A_{pt}} \rho_n \tau_n + PT_{cn} \frac{A_f}{A_n} \rho_n \tau_n - PT_c \frac{A_c}{A_{pt}} \rho_p \tau_{pt-p} + P_c \frac{A_c}{A_p} \rho_p \tau_{pt-p}$$
$$- PT_c \frac{A_c}{A_{pt}} \rho_{np} \tau_{pt-np} + NP_c \frac{A_c}{A_{np}} \rho_{np} \tau_{pt-np} - PT_c \frac{A_c}{A_{pt}} \rho_{sk} \tau_{hcw-pt} f(t)$$
$$+ HCW \frac{A_c}{A_{hcw}} \rho_{sk} \tau_{hcw-pt} f(t) - PT_c \mu_{sk} \tag{1}$$

where, m∈Z+, and

$$f(t) = \begin{cases} 1, & t \in [m-1, m-\frac{2}{3}) \\ 0, & t \in \left[m-\frac{2}{3}, m\right) \end{cases} \tag{2}$$

The function $f(t)$ is a time indicator function for a HCW's visit in the colonized patient's room. $f(t)$ was equal to one during the first 20 minutes, allowing HCW touching events to occur and equal to 0 during other times.

PT_c was initialized at the equilibrium MRSA level of 6,000 cfu/2000 cm^2.

2) The porous surface in the colonized patient's room (P_c)

Changes in MRSA concentration on the porous surface in the colonized patient's room (P_c), as described in Equation 3, were driven by the deposition of MRSA on the surface, surface touches by the colonized patient, surface touches by the HCWs during the first 20 minutes of the hour, natural die-off, and daily surface decontamination.

$$\frac{dP_c}{dt} = \alpha A_p - P_c \frac{A_c}{A_p} \rho_p \tau_{pt-p} + PT_c \frac{A_c}{A_{pt}} \rho_p \tau_{pt-p} - P_c \frac{A_c}{A_p} \rho_p \tau_{hcw-p} f(t)$$
$$+HCW \frac{A_c}{A_{hcw}} \rho_p \tau_{hcw-p} f(t) - P_c \epsilon_d h(t) - P_c \mu_p \tag{3}$$

where, m∈Z+, and

$$h(t) = \begin{cases} 1, & t = m * 24 \\ 0, & otherwise \end{cases} \tag{4}$$

The function $h(t)$ is a time indicator function that regulates decontamination to every 24 hours.

3) The nonporous surface in the colonized patient's room (NP_c)

Changes in MRSA concentration on the nonporous surface in the colonized patient's room (NP_c), as described in Equation 5, were driven by the deposition of MRSA dispersal on the

surface, surface touches by the colonized patient, surface touches by the HCWs during the first 20 minutes of the hour, the natural die-off, and the daily surface decontamination. Structurally, the nonporous surface is similar to the porous surface, except that only the nonporous surfaces can be wiped off following a HCW touch. The wiping rate is as frequent as the rate at which HCWs touch the nonporous surface. The efficacy of the wipes and the wiping rate is denoted by ε_w and ω_{hcw-np}, respectively.

$$\frac{dNP_c}{dt} = \alpha A_{np} - NP_c \frac{A_c}{A_{np}} \rho_{np} \tau_{pt-np} + PT_c \frac{A_c}{A_{pt}} \rho_{np} \tau_{pt-np} - NP_c \frac{A_c}{A_{np}} \rho_{np} \tau_{hcw-np} f(t)$$
$$+HCW \frac{A_c}{A_{hcw}} \rho_{np} \tau_{hcw-np} f(t) - NP_c \frac{A_c}{A_{np}} \epsilon_w \omega_{hcw-np} f(t) - NP_c \epsilon_d h(t) - NP_c \mu_{np} \quad (5)$$

4) The uncolonized patient (PT_u)

Changes in MRSA concentration on the skin and hands of the uncolonized patient (PT_u), as described in Equation 6, were driven by contacts with HCWs during the second 20 minutes of the hour, contacts with the two room surfaces, contact with one's own nose, and the natural die-off on the skin and hand.

$$\frac{dPT_u}{dt} = -PT_u \frac{A_f}{A_{pt}} \rho_n \tau_n + PT_{un} \frac{A_f}{A_n} \rho_n \tau_n - PT_u \frac{A_c}{A_{pt}} \rho_p \tau_{pt-p} + P_u \frac{A_c}{A_p} \rho_p \tau_{pt-p}$$
$$-PT_u \frac{A_c}{A_{pt}} \rho_{np} \tau_{pt-np} + NP_u \frac{A_c}{A_{np}} \rho_{np} \tau_{pt-np} - PT_u \frac{A_c}{A_{pt}} \rho_{sk} \tau_{hcw-pt} g(t) + HCW \frac{A_c}{A_{hcw}} \rho_{sk} \tau_{hcw-pt} g(t)$$
$$-PT_u \mu_{sk} \quad (6)$$

where, m∈Z+, and

$$g(t) = \begin{cases} 1, & t \in [m - \frac{2}{3}, m - \frac{1}{3}) \\ 0, & t \in \left[m - 1, m - \frac{2}{3}\right) or [m - \frac{1}{3}, m] \end{cases} \quad (7)$$

The function $g(t)$ is a time indicator function regulating the HCW's visit in the uncolonized patient's room. $g(t)$ was equal to one during the second 20 minutes of the hour, allowing the HCW's touching events to occur and equals 0 during other times.

5) The porous surface in the uncolonized patient's room (P_u)

Changes in MRSA concentration on the porous surface in the uncolonized patient's room, as described in Equation 8, were similar to those in the porous surface in the colonized patient's room, except that there was no MRSA dispersal and deposition in the uncolonized patient's room. Surface touches by the HCW occurred during the second 20 minutes of the hour.

$$\frac{dP_u}{dt} = -P_u \frac{A_c}{A_p} \rho_p \tau_{pt-p} + PT_u \frac{A_c}{A_{pt}} \rho_p \tau_{pt-p} - P_u \frac{A_c}{A_p} \rho_p \tau_{hcw-p} g(t) + HCW \frac{A_c}{A_{hcw}} \rho_p \tau_{hcw-p} g(t) - P_u \epsilon_d h(t) - P_u \mu_p \quad (8)$$

6) The nonporous surface in the uncolonized patient's room (NP_u)

Changes in MRSA concentration on the nonporous surface in the uncolonized patient's room, as described in Equation 9, were similar to those in the nonporous surface in the colonized patient's room, except that there was no MRSA dispersal and deposition in the uncolonized patient's room. Surface touches by the HCW occurred during the second 20 minutes of the hour.

$$\frac{dNP_u}{dt} = -NP_u \frac{A_c}{A_{np}} \rho_{np} \tau_{pt-np} + PT_u \frac{A_c}{A_{pt}} \rho_{np} \tau_{pt-np} - NP_u \frac{A_c}{A_{np}} \rho_{np} \tau_{hcw-np} g(t) + HCW \frac{A_c}{A_{hcw}} \rho_{np} \tau_{hcw-np} g(t) - NP_u \frac{A_c}{A_{np}} \epsilon_w \omega_{hcw-np} g(t) - NP_u \epsilon_d h(t) - NP_u \mu_{np} \quad (9)$$

7) The healthcare workers (HCWs)

Changes in MRSA concentration on the exposed skin and hands of HCWs, as described in Equation 10, were driven by all HCW activities and natural die-off on skin and hands.

Activities of HCWs included touching the colonized patient and the room surfaces during the first 20 minutes while in the colonized patient's room, touching the uncolonized patient and the room surfaces during the second 20 minutes while in the uncolonized patient's room, and touching one's own noses. HCWs may also wipe the nonporous surfaces after a nonporous surface touch. HCWs were assumed to have clean skin and hands at the beginning of each 8-hour shift. The time indicator for the beginning of the shift is *s(t)*.

$$\frac{dHCW}{dt} = -HCW\frac{A_f}{A_{hcw}}\rho_n\tau_n + HCW_n\frac{A_f}{A_n}\rho_n\tau_n - HCW\frac{A_c}{A_{hcw}}\rho_{sk}\tau_{hcw-pt}f(t)$$
$$+PT_c\frac{A_c}{A_{pt}}\rho_{sk}\tau_{hcw-pt}f(t) - HCW\frac{A_c}{A_{hcw}}\rho_p\tau_{hcw-p}f(t) + P_c\frac{A_c}{A_p}\rho_p\tau_{hcw-p}f(t)$$
$$-HCW\frac{A_c}{A_{hcw}}\rho_{np}\tau_{hcw-np}f(t) + NP_c\frac{A_c}{A_{np}}\rho_{np}\tau_{hcw-np}f(t) - HCW\frac{A_c}{A_{hcw}}\rho_{sk}\tau_{hcw-pt}g(t)$$
$$+PT_u\frac{A_c}{A_{pt}}\rho_{sk}\tau_{hcw-pt}g(t) - HCW\frac{A_c}{A_{hcw}}\rho_p\tau_{hcw-p}g(t) + P_u\frac{A_c}{A_p}\rho_p\tau_{hcw-p}g(t)$$
$$-HCW\frac{A_c}{A_{hcw}}\rho_{np}\tau_{hcw-np}g(t) + NP_u\frac{A_c}{A_{np}}\rho_{np}\tau_{hcw-np}g(t) - HCWs(t) - HCW\mu_{sk}$$

(10)

where, m∈Z+ and

$$s(t) = \begin{cases} 1, & t = m * 8 \\ 0, & otherwise \end{cases}$$

(11)

The other two compartments in the model are MRSA accumulated in the uncolonized patient's nose and HCW's nose. They are given by the following equations:

$$\frac{dPT_{un}}{dt} = -PT_{un}\frac{A_f}{A_n}\rho_n\tau_n + PT_u\frac{A_f}{A_{pt}}\rho_n\tau_n$$

(12)

$$\frac{dHCW_n}{dt} = -HCW_n\frac{A_f}{A_n}\rho_n\tau_n + HCW\frac{A_f}{A_{hcw}}\rho_n\tau_n$$

(13)
