# Supplementary material for: The dynamics of methicillin-resistant Staphylococcus aureus exposure in a hospital model and the potential for environmental intervention
Source: BMC Infect Dis. 2013 Dec 17;13:595. doi: 10.1186/1471-2334-13-595 (PMC3878576; doi:10.1186/1471-2334-13-595)
Supplement: Additional file 3: Supplementary material III — Sensitivity analysis. [file 1471-2334-13-595-S3.docx]

Supplemental material III

**Sensitivity Analysis**

To understand how our model parameter values affect the inferences resulting from our analysis, we examined the sensitivity of the exposure dose to HCWs and the uncolonized patient with varying transfer efficiency, survivability, and HCW contact rate parameters. In all these sensitivity analyses, direct exposure referred to exposure from the colonized patient to HCWs and exposure from HCWs to the uncolonized patient, whereas indirect exposure referred to exposure from both porous and nonporous surfaces to HCWs and exposure from both surfaces to the uncolonized patient.

Transfer efficiency and pathogen survivability are the main two parameters that differentiate the type of surfaces we modeled, i.e., porous and nonporous. Since our simulations suggested that nonporous surfaces are the dominant exposure source to HCWs and the uncolonized patient, we chose to examine how this dominance might be affected if the nonporous surface transfer efficiency (ρ_np_) values varied anywhere between 0.05 and 0.75 (a value of 0.4 was used in the main simulation analysis). Our outcome exposure variables were not sensitive to changes in ρ_np_. Even at the lower bound, where ρ_np_ = 0.05, indirect exposure to HCWs in the colonized patient’s room remained the dominant source of exposure (Figure S1). Exposure to the uncolonized patient, however, was dominated by direct exposure to the HCW for lower values of ρ_np_, but as ρ_np_ approached 0.75, exposure was split evenly between direct and indirect (Figure S2). Exposure was even more sensitive to skin transfer efficiency values. The indirect exposure to the HCWs remained dominant regardless of the level of skin transfer efficiency, but for low values, direct exposure to the colonized patient became negligible (Figure S3). Likewise, for the uncolonized patient, the indirect exposure was not dominant when the skin transfer efficiency was high but became dominant for lower values (Figure S4).

Survivability describes how quickly the pathogens die off from surfaces or hands. We examined the sensitivity due to both die-off rates by taking the following steps: (1) assigning a range of the nonporous surface die-off rate (μ_np_) between 1.6 × 10^-5^ and 1.6 × 10^-3^ min^-1^, where μ_np,_ was assigned a value of 2 × 10^-4^ in the main simulation; and (2) assigning a range of the skin die-off rate (μ_sk_) between 1 × 10^-4^ and 1 × 10^-2^ min^-1^, where μ_sk_ was assigned a value of 3.53 × 10^-3^ in the main simulation. The exposure to the HCW was not very sensitive to the range of nonporous die-off rate used (Figure S5), and the exposure to the uncolonized patient was only somewhat more sensitive (Figure S6). Exposure to either the HCW or uncolonized patients was more sensitive to the skin die-off rates explored, although none of the values resulted in changes in our qualitative conclusions (Figures S7 and S8). Interestingly, for very low skin die-off rates, the exposure to the uncolonized patient began to decrease because their contamination levels were high compared to the contamination levels of the surfaces.

To examine how a human behavioral factor may affect exposure, we examined a range of HCW’s surface and patient contact from 3 to 19 times per hour. In the main simulation, HCWs touched each patient and each surface 8 times per hour. Again, although we observed some variation in exposure levels, the dominant exposure sources (indirect versus direct) remain the same (Figures S9 and S10).

To further explore the variable nature of contact rates, we compared a highly touched nonporous surface (2.5 times higher than baseline) with a minimally touched nonporous surface (0.5 times baseline) in terms of its impact on exposures with and without an intervention (Figures S11 and S12). The highly touched surface was a much larger source of exposure compared to the minimally touched surface. Surface wiping appeared to be superior to daily decontamination in decreasing the exposure dose, primarily due to its increased frequency. Again, since our model structure did not allow for the exploration of spatial heterogeneity, the spatially specific nature of surface wiping was a feature that would provide additional benefits.

Finally, we performed a multivariate sensitivity analysis to examine the potential joint effects of nonporous surface transfer efficiency, skin die-off rate and HCW’s contact rate. By comparing all the combinations of a high and low value, the results summarized in Table S3 suggest that each parameter roughly contributed to an independent effect to exposure. As the number of variables with high values increased, the exposure increased. Skin survivability seemed to have the weakest effect and nonporous transfer efficiency, the strongest effect on exposure.

**Figure S1.** The proportion of the HCW’s indirect exposure in the colonized patient’s room as a function of the nonporous surface transfer efficiency. Indirect exposure is the sum exposure from the porous and nonporous surfaces to the HCWs. Percentage of indirect exposure to the HCWs is shown above each bar. Simulation analysis in the main manuscript used 0.4 as the nonporous surface transfer efficiency, resulting in 70% indirect exposure to the HCWs in the colonized patient’s room.

**Figure S2.** The proportion of the uncolonized patient’s indirect exposure as a function of the nonporous surface transfer efficiency. Indirect exposure is the sum exposure from the porous and nonporous surfaces to the uncolonized patient. Percentages of indirect exposure to the uncolonized patient are shown above each bar. Simulation analysis in the main manuscript used 0.4 as the nonporous surface transfer efficiency, resulting in 35% indirect exposure to the uncolonized patient.

**Figure S3.** The proportion of the HCW’s indirect exposure in the colonized patient’s room as a function of the skin transfer efficiency. Indirect exposure is the sum exposure from the porous and nonporous surfaces to the HCW. Percentage of indirect exposure to the HCWs is shown above each bar. Simulation analysis in the main manuscript used 0.35 as the skin transfer efficiency, resulting in 70% indirect exposure to the HCWs in the colonized patient’s room.

**Figure S4.** The proportion of the uncolonized patient’s indirect exposure as a function of the skin transfer efficiency. Indirect exposure is the sum exposure from the porous and nonporous surfaces to the uncolonized patient. Percentages of indirect exposure to the uncolonized patient are shown above each bar. Simulation analysis in the main manuscript used 0.35 as the skin transfer efficiency, resulting in 35% indirect exposure to the uncolonized patient.

**Figure S5.** The proportion of the HCW’s exposure in the colonized patient’s room that is indirect as a function of the nonporous surface die-off rate. Indirect exposure is the sum exposure from the porous and nonporous surfaces to the HCW. Percentage of indirect exposure to the HCWs is shown above each bar. Simulation analysis in the main manuscript used 2 × 10^-4^ min^-1^ as the nonporous surface die-off rate, resulting in 70% indirect exposure to the HCWs in the colonized patient’s room.

**Figure S6.** The proportion of the uncolonized patient’s indirect exposure as a function of the nonporous surface die-off rate. Indirect exposure is the sum exposure from the porous and nonporous surfaces to the uncolonized patient. Percentages of indirect exposure to the uncolonized patient are shown above each bar. Simulation analysis in the main manuscript used 2 × 10^-4^ min^-1^ as the nonporous surface die-off rate, resulting in 35% indirect exposure to the uncolonized patient.

**Figure S7.** The proportion of the HCW’s indirect exposure in the colonized patient’s room as a function of the skin die-off rate. Indirect exposure is the sum exposure from the porous and nonporous surfaces to the HCW. Percentage of indirect exposure to the HCWs is shown above each bar. Simulation analysis in the main manuscript used 35.3 × 10^-4^ min^-1^ as the skin die-off rate, resulting in 70% indirect exposure to the HCWs in the colonized patient’s room.

**Figure S8.** The proportion of the HCW’s indirect exposure as a function of the skin die-off rate. Indirect exposure is the sum exposure from the porous and nonporous surfaces to the uncolonized patient. Percentages of indirect exposure to the HCW are shown above each bar. Simulation analysis in the main manuscript used 35.3 × 10^-4^ min^-1^ as the skin die-off rate, resulting in 35% indirect exposure to the HCW.

**Figure S9.** The proportion of the HCW’s indirect exposure as a function of the HCW’s contact rate. The HCW’s contact rate of surfaces and of patients are assumed to be equal. Indirect exposure is the sum exposure from the porous and nonporous surfaces to the HCW. Percentages of indirect exposure to the uncolonized patient are shown above each bar. Simulation analysis in the main manuscript used 8 as the number of times the HCW touches each surface and patient per visit, resulting in 70% indirect exposure to the uncolonized patient.

**Figure S10.** The proportion of the uncolonized patient’s indirect exposure as a function of the HCWs’ contact rate. The HCW’s contact rates of surfaces and of patients are assumed to be equal. Indirect exposure is the sum exposure from the porous and nonporous surfaces to the uncolonized patient. Percentages of indirect exposure to the uncolonized patient are shown above each bar. Simulation analysis in the main manuscript used 8 as the number of times the HCW touches each surface and patient per visit, resulting in 35% indirect exposure to the uncolonized patient.

**Figure S11.** Effects of daily decontamination and surface wiping in decreasing exposure to HCWs. Three types of exposure are shown here: (1) exposure from the colonized patient, (2) exposure from minimally touched nonporous surface, and (3) exposure from highly touched nonporous surface. The numbers at the top of each bar are the percent reduction following the intervention compared to when there was no intervention.

**Figure S12.** Effects of daily decontamination and surface wiping in decreasing exposure to the uncolonized patient. Three types of exposure are shown here: (1) exposure from HCWs, (2) exposure from minimally touched nonporous surface, and (3) exposure from highly touched nonporous surface. The numbers at the top of each bar are the percent reduction following the intervention compared to when there was no intervention.

**Table S3.** Multivariate sensitivity analysis of nonporous surface transfer efficiency, skin survivability, and HCW’s contact rate to surfaces and patients. These three parameters take on high or low values. The nonporous transfer efficiency (ρ_np_ ) is either 0.1 (low) or 0.6 (high). The skin die-off rate (µ_sk_) is either 1 × 10^-4^ min^-1^(low) or 1 × 10^-2^ min^-1^(high). The HCW’s contact rate (τ_hcw-pt_, τ_hcw-sf_) is either 2 times per visit (low) or 20 times per visit (high). The outcomes are indirect exposure levels to the HCW, percentage of indirect exposure to the HCW, indirect exposure levels to the uncolonized patient, and percentage of indirect exposure to the uncolonized patient. The color scales rank exposure levels (orange = highest exposure, yellow = lowest exposure).

| Nonporous transfer efficiency | low | low | low | low | high | High | high | high |
| --- | --- | --- | --- | --- | --- | --- | --- | --- |
| Skin survivability | low | low | high | high | low | Low | high | high |
| HCW’s contact rate | low | high | low | high | low | high | low | high |
| Indirect exposure  to HCW | 629.33 | 1254.57 | 417.39 | 1609.3 | 1371.7 | 1541.44 | 649.4 | 1819.26 |
| % Indirect exposure  to HCW | 37 | 49 | 70 | 81 | 65 | 61 | 79 | 85 |
| Indirect exposure  to uncolonized patient | 0 | 0.58 | 8.19 | 54.38 | 80.62 | 50.78 | 55.12 | 198.1 |
| % Indirect exposure  to uncolonized patient | 0 | 0 | 20 | 12 | 35.4 | 15.47 | 59 | 37 |
